# Acute doses of caffeine shift nervous system cell expression profiles toward promotion of neuronal projection growth

**DOI:** 10.1038/s41598-017-11574-6

**Published:** 2017-09-13

**Authors:** Nancy Y. Yu, Andrea Bieder, Amitha Raman, Enrichetta Mileti, Shintaro Katayama, Elisabet Einarsdottir, Bertil B. Fredholm, Anna Falk, Isabel Tapia-Páez, Carsten O. Daub, Juha Kere

**Affiliations:** 1 Department of Biosciences and Nutrition, Karolinska Institutet, Huddinge, SE-141 83 Sweden; 2 Department of Medicine (MedH), Karolinska Institutet, Huddinge, SE-141 86 Sweden; 3 Folkhälsan Institute of Genetics, and Molecular Neurology Research Program, University of Helsinki, Helsinki, 00014 Finland; 4 Department of Physiology and Pharmacology, Karolinska Institutet, Solna, SE-171 77 Sweden; 5 Department of Neuroscience, Karolinska Institutet, Solna, SE-171 77 Sweden; 6 Division of Genomic Technologies, RIKEN Center for Life Science Technologies, Tsurumi, Yokohama, Kanagawa #230-0045 Japan; 7 Department of Medical and Molecular Genetics, King’s College London, London, SE1 9RT United Kingdom; 8 Present Address: Department of Medicine/Center for Molecular Medicine, Karolinska University Hospital, Solna, SE-171 76 Sweden

## Abstract

Caffeine is a widely consumed psychoactive substance, but little is known about the effects of caffeine stimulation on global gene expression changes in neurons. Here, we conducted gene expression profiling of human neuroepithelial stem cell-derived neurons, stimulated with normal consumption levels of caffeine (3 μM and 10 μM), over a period of 9 h. We found dosage-dependent activation of immediate early genes after 1 h. Neuronal projection development processes were up-regulated and negative regulation of axon extension processes were down-regulated at 3 h. In addition, genes involved in extracellular matrix organization, response for wound healing, and regulation of immune system processes were down-regulated by caffeine at 3 h. This study identified novel genes within the neuronal projection guidance pathways that respond to acute caffeine stimulation and suggests potential mechanisms for the effects of caffeine on neuronal cells.

## Introduction

Caffeine, the principal alkaloid in coffee, tea, and energy drinks, is one of the most consumed psychoactive substances in the world^1^. Studies suggest that coffee consumption affects health-related variables such as cancer^2, 3^, exercise performance^4^, diabetes^5^, and blood pressure^6^. Caffeine has been shown to affect multiple aspects of the central nervous system, and to influence e.g. cognitive performance^7^, memory improvement^8^, mood improvement^9^, increased alertness^10, 11^, increase in overall metabolism in the brain^1, 12^, changes in dopaminergic transmission^13^, and motor neuron stimulation^14^. Regular consumption of coffee/caffeine has been linked with possible protection against cognitive decline^15, 16^, especially Parkinson’s disease^1, 17, 18^. On the other hand, caffeine also negatively affects sleep quality^1^, and may increase anxiety in sensitive individuals^19^.

At a concentration of 1–30 μM in the body (equivalent to recent ingestion of 1–5 cups of coffee^1^), the primary effect of caffeine in the central nervous system is inhibition of adenosine receptors and subsequent modulation of neurotransmitter release^1, 12, 20, 21^. Adenosine, a neuro-modulatory signaling molecule, is normally present in the brain, and when it accumulates e.g. during increased neuronal firing, it causes a progressive decrease in neuronal activity when bound to adenosine receptors. Caffeine counters this effect by acting as an antagonist at the adenosine receptors A_1_ and A_2A_^1^. By preventing adenosine from binding, caffeine increases neuronal activity, leading to downstream stimulatory effects on the neurons. At supraphysiological concentrations (>100 μM), caffeine inhibits GABA_A_ receptors, reducing the inhibitory input in functional neuronal networks^22^, inhibits phosphodiesterase activity leading to increased cellular cAMP levels^23^, and releases Ca^2+^ from intracellular ryanodine sensitive stores stimulating Ca^2+^ signaling in numerous cell types including neurons^24–26^.

At the gene-regulatory level, caffeine modulates CREB-dependent gene expression and induces immediate-early genes (IEGs)^27, 28^. IEGs are transiently expressed and have long been used as biomarkers for neuronal activation^29^. Caffeine has been shown to increase the expression of IEGs including JunB, c-Jun, AP-1, c-Fos and preproenkephalin (PENK)^28^. In terms of global gene expression, a microarray study on adenosine A_2A_ receptor knock out mice found that genes involved in adipocyte differentiation/insulin signaling pathway were enriched in the mouse striatum from caffeine treatment^30^.

Previous research on the effects of caffeine on neuronal cells has been limited to animal models, animal primary cells, or human cancer cell lines. This limitation can be overcome by using neurons derived from human long-term self-renewing neuroepithelial stem cells (lt-NES cells, here termed NES cells). NES cells are self-renewing in the presence of fibroblast growth factor﻿ (FGF) and epidermal growth factor (EGF) and differentiate into neurons and glia upon growth factor removal^31, 32^. They have been successfully used as a model to investigate neural developmental processes and disorders^33–36^.

Transcriptomic analysis allows monitoring of genome wide expression changes rather than measuring expression of single genes. Cap Analysis of Gene Expression (CAGE) is a sensitive and quantitative RNA sequencing method that captures the ultimate 5′ transcription start sites, allowing the annotation of transcriptome changes at the promoter level rather than at the gene level only^37^. It has been used previously to quantify promoter-specific transcripts in order to generate a comprehensive mammalian gene expression atlas^38^.

In contrast to abundant research on epidemiology and pharmacology, there is very little available data exploring the effects of caffeine at the whole-cell regulatory network level, utilizing modern molecular biology approaches. To our knowledge, the effects of caffeine in consumption doses on global gene expression changes in human neurons have not yet been examined. We therefore sought to study gene expression changes upon caffeine stimulation in a human NES cell-derived neuronal model using high-throughput transcriptome profiling.

## Results

### Differentiation and phenotyping of NES-derived neuronal cell cultures

**Figure 1:**
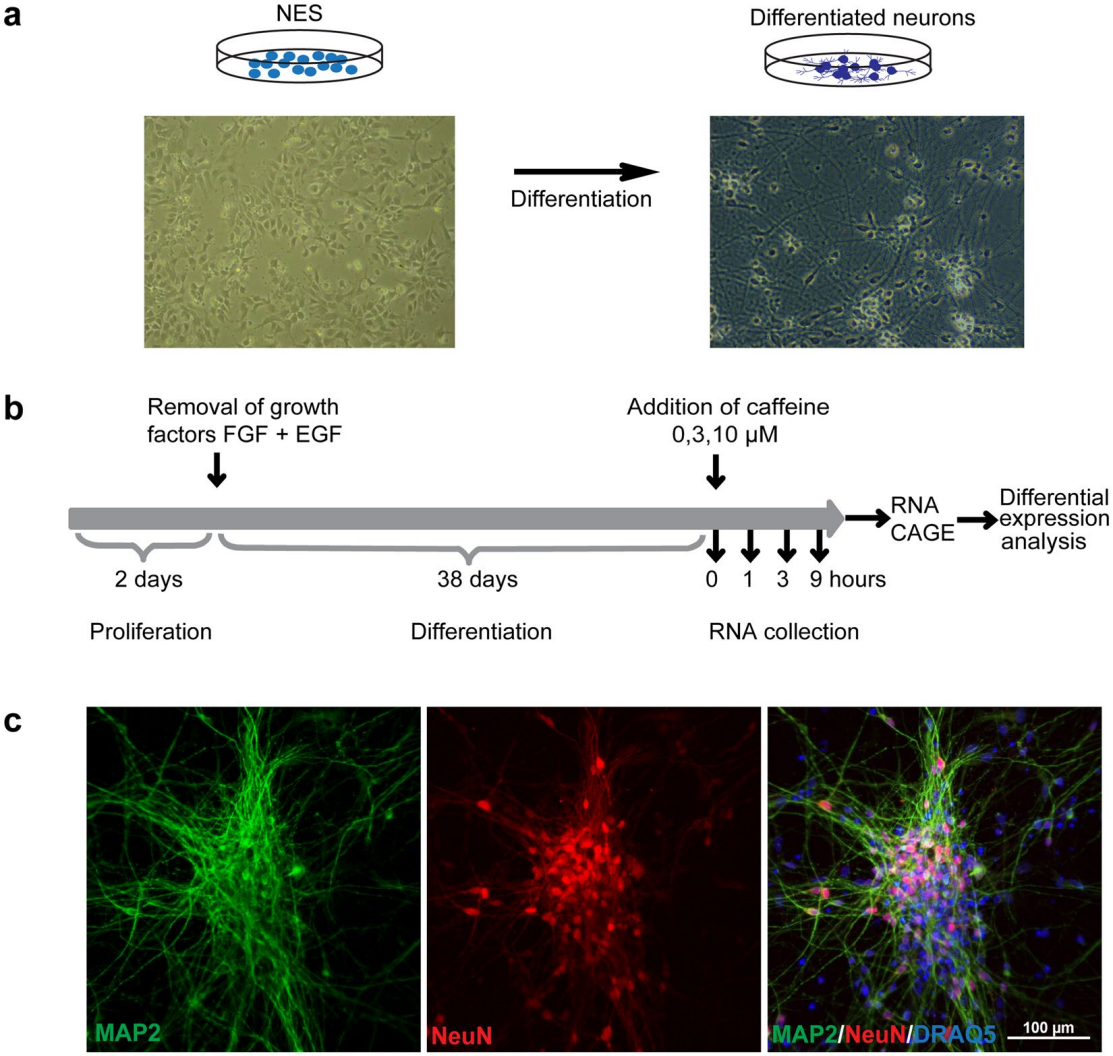
Experimental design. (**a**) NES cell differentiation: bright field images of NES cells and differentiated neurons. (**b**) Timeline of the experiment. NES cells were plated and kept in the presence of FGF and EGF for two days. Growth factors were removed and cells were differentiated for 38 days. Caffeine was added and RNA was collected after 0 h, 1 h, 3 h and 9 h. The samples were then used for CAGE sequencing and differential expression analysis. (**c**) Phenotype of differentiated cells. After 38 days, cells were fixed and stained for the neuronal markers MAP2 and NeuN and the nuclear marker DRAQ5. Untreated cells are shown.

We differentiated NES cells during 38 days by removal of the growth factors FGF and EGF from the cell medium (Fig. 1). At this stage, the cells were differentiated predominantly into mature neurons expressing MAP2 and NeuN (Fig. 1c), with approximately 10% of the cells being glial cells expressing GFAP (Supplementary Figure S1). We then changed to medium containing caffeine at three different concentrations (0 μM, 3 μM and 10 μM) and collected RNA after the indicated time points. At the latest time point, we did not observe any visible changes in overall morphology between caffeine-treated and non-treated cells by inspection with bright field microscope (data not shown).

### Adenosine receptors A_1_ and A_2B_ are the dominant receptors expressed in the neuronal cell cultures

Since caffeine affects neurons mainly through antagonizing adenosine receptors, we examined the gene expression levels of all genes within the adenosine receptor family: A_1_ (ADORA1), A_2A_ (ADORA2A), A_2B_ (ADORA2B), and A_3_ (ADORA3). Two isoforms of A_1_ were expressed throughout the time course, one at ~20 tags per million (TPM) and the other at 0.5 TPM. A_2B_ was expressed at ~10 TPM (Supplementary Figure S2). The expression levels of the adenosine receptor genes were not affected by caffeine. Expression of A_2A_ and A_3_ was not detected in our samples.

### Overall differential gene expression is perturbed in a time-dependent manner with more genes down-regulated than up-regulated by caffeine

**Figure 2:**
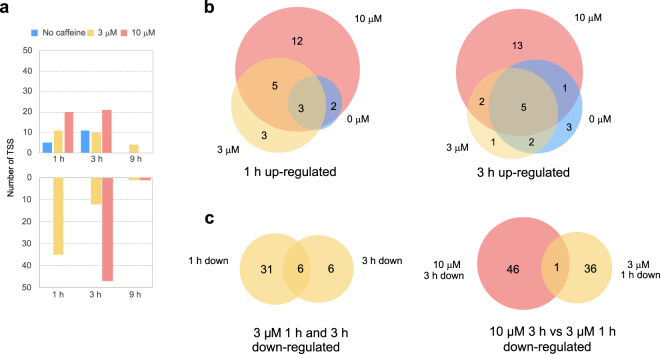
(**a**) Numbers of up- and down-regulated genes at each time point compared to no caffeine control at 0 h. (**b**) Venn diagrams showing numbers of overlapping genes with different caffeine dosages up-regulated after 1 h (left) and 3 h (right). (**c**) Venn diagrams showing overlaps of down-regulated genes between 3 μM caffeine at 1 h vs. 3 h (left), and between 3 μM caffeine at 1 h vs. 10 μM caffeine at 3 h (right).

We asked how caffeine stimulation affects gene expression in a time- and concentration- dependent manner. We therefore performed differential expression analysis and compared gene expression from different concentrations of caffeine at different time points to control at time 0 (no caffeine added). The number of up-regulated genes compared to control increased in a caffeine dosage-dependent manner after 1 h and 3 h (Fig. 2a). The up-regulated genes between no caffeine, 3 μM and 10 μM caffeine treatments largely overlapped (Fig. 2b, Supplementary Table S1). The expression of these genes mostly returned to baseline level by 9 h.

The number of down-regulated genes in caffeine-treated samples after 1 h and 3 h was higher than the number of up-regulated genes. After 1 h, 37 transcription start sites (TSS), corresponding to 34 genes, were down-regulated by 3 μM caffeine, and the number of down-regulated genes decreased after 3 ﻿h﻿ and 9 h (Fig. 2a, Table S1). With 10 μM caffeine, 47 TSS (45 genes) were down-regulated at 3 h, with the majority of the gene expression levels returning to baseline by 9 h (Fig. 2a). We asked if the genes down-regulated at 1 h and 3 h in the 3 μM caffeine-treated samples were the same genes; 50% of the TSS from the 3 h gene list overlapped with TSS from 1 h (Fig. 2c, Table S1). We also asked if the genes down-regulated at 3 h in the 3 μM caffeine-treated samples were in common with the down-regulated TSS in the 10 μM samples, and found only 1 TSS in common between these two gene sets (Fig. 2c, Table S1). In general, caffeine induced more down-regulation than activation of genes, where similar genes were activated by different caffeine dosages, but different genes were down-regulated by different caffeine dosages at each time point.

### Immediate-Early Response genes are up-regulated with caffeine addition

**Figure 3:**
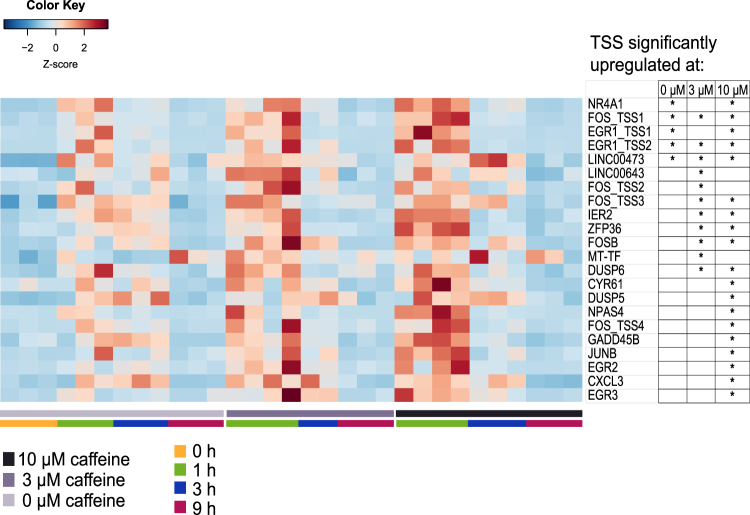
Heatmap of relative expression levels of TSS up-regulated at 1 h for all caffeine concentrations. The relative expressions for all time points are shown for these TSS. An asterisk marks the TSS that are significantly up-regulated at the respective caffeine concentrations (adjusted p < 0.05).

Next, we asked which gene classes were differentially up-regulated upon caffeine stimulation. Among all the TSS up-regulated at 1 h (25 TSS combining all the conditions), 20 of the TSS mapped to genes belonging to the immediate early response gene family (IEG). In addition, 6 of the 27 TSS up-regulated at 3 h were also in the IEG family (Table S1). IEGs are genes that are activated transiently and rapidly in response to a wide variety of cellular stimuli^39^. Our results showed that overall, the higher the caffeine dosages, the more IEGs were activated with greater expression intensity compared to control (Fig. 3). Two other TSS up-regulated at 1 h that were not part of the reference IEG list belonged to long non-coding RNAs, LINC00473 and LINC00643, also shown in Fig. 3. LINC00473 (C6orf176) has been implicated to play a role in cAMP-mediated gene expression^40^.

### Neuronal projection genes are differentially expressed with higher doses of caffeine

**Figure 4:**
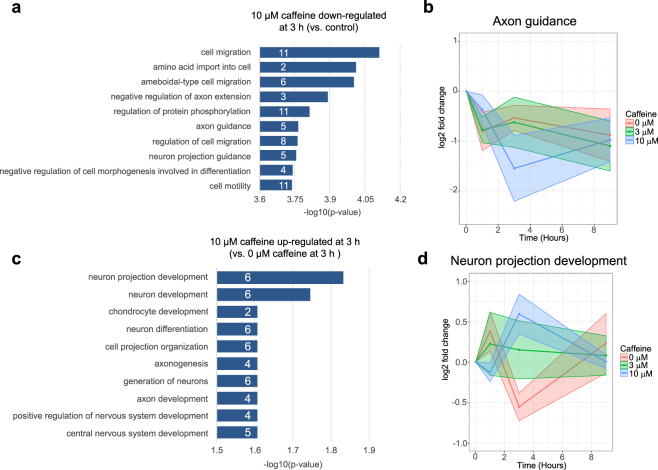
(**a**) GO enrichment of biological processes down-regulated by 10 μM caffeine treatment after 3 h. Each bar depicts the −log 10 (adjusted p-value) for each GO term. The number of genes that belong to each GO biological process is marked in white on each bar. (**b**) Time course progression plot showing mean log2 fold change values for genes belonging to the axon guidance process. The x-axis corresponds to time in hours and the y-axis corresponds to log2 fold change compared to control at time 0. (**c**) GO enrichment of biological processes for genes up-regulated at hour 3 by 10 μM caffeine compared to 0 μM and 3 μM caffeine treatment. (**d**) Time course progression plot showing mean log2 fold change values for neuronal projection development genes with dosage-dependent up-regulation at 3 h.

To further elucidate the gene classes that were differentially expressed upon caffeine exposure, we performed GO enrichment analysis. Among 45 genes (47 TSS) significantly down-regulated after 3 h of 10 μM caffeine treatment (adjusted p < 0.05), top enriched GO biological processes including cell migration (GO:0016477), negative regulation of axon extension (GO:0030517), axon guidance (GO:0007411), and regulation of protein phosphorylation (GO:0001932) (Fig. 4a, Supplementary Table S3). Many genes in the axon/neuronal projection guidance categories (NTN1, SEMA4B, SEMA5B, EFNA1, EFNA4) are also included in the cell migration/cell motility categories. Figure 4b shows a time-course progression plot of the average fold change of axon guidance gene expressions at each time point (compared to time 0 control). Overall time course progression for the axon guidance genes showed more similar progression between 3 μM caffeine treatment and 0 μM caffeine treatment whereas the gene expression fluctuation for 10 μM caffeine treatment showed a more distinct progression (Fig. 4b).

A second group of neuronal development processes-related genes was enriched among the gene set up-regulated at 3 h by 10 μM caffeine treatment compared to 0 μM and 3 μM caffeine treatment (Fig. 4c). 16 genes (18 TSS) were up-regulated, 6 of which are involved in neuronal projection development (Supplementary Table S2). The time course progression plot showed that the mean gene expression fold change levels for these neuronal projection development genes for 3 μM were closer to 0 μM caffeine treatment at 1 h and in between 0 μM and 10 μM caffeine treatment at 3 h (Fig. 4d).

### Extracellular matrix organization and immune system regulation genes are down-regulated transiently with higher doses of caffeine

**Figure 5:**
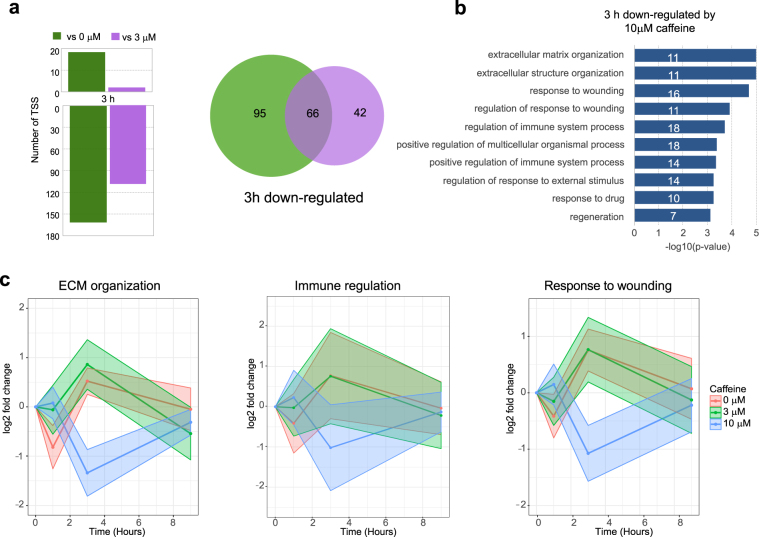
(**a**) Bar plots showing dosage-dependent up and down-regulated TSS at 3 h (left). Venn diagram showing overlap between the number of TSS down-regulated by 10 μM vs. 0 μM caffeine and the number of genes down-regulated by 10 μM caffeine vs. 3 μM caffeine treatment. (**b**) GO enrichment results for the 66 overlapping 3 h dosage-dependent caffeine response genes. Each bar depicts the −log 10 (adjusted p-value) for each GO term. The number of genes that belong to each GO biological process is marked in white on each bar. (**c**) Time course progression plots showing mean log2 fold change values for genes involved in extracellular matrix organization, regulation of immune system process, and response to wounding. The x-axis corresponds to time in hours and the y-axis corresponds to log2 fold change compared to control at time 0.

In order to exclude the time-dependent component of the effect on expression, we compared samples treated with different doses of caffeine at the same time points. No genes were differentially expressed at 1 h or 9 h in a dosage-dependent manner. However, at 3 h, 161 TSS (140 genes) were down-regulated at 3 h by 10 μM caffeine treatment compared to 0 μM caffeine treatment (Fig. 5a, Supplementary Table S3). 108 TSS (96 genes) were significantly down-regulated by the 10 μM caffeine treatment compared to 3 μM caffeine treatment (Fig. 5a). 45% of these genes (62 genes, or 66 TSS) overlapped between these 2 comparisons, suggesting that these genes have dosage-dependent responses. GO enrichment analysis of the 62 genes identified extracellular matrix organization (GO:0030198), response to wounding (GO:0009611), and regulation of immune system (GO:0002682) as biological process perturbed by 10 μM caffeine treatment (Fig. 5b). Averaged time course progression plots for these 3 processes revealed that overall, 3 μM caffeine treatment behaved more similarly to 0 μM caffeine treatment than to 10 μM caffeine treatment (Fig. 5c).

## Discussion

To our knowledge, this is the first study of caffeine-induced perturbation of whole transcriptome gene expression changes in a human neuronal cell model, here differentiated from NES cells, that most resemble natural human central nervous system cells. Our differentiated cell cultures consisted of glial cells and neurons, but were not optimized specifically to be enriched for particular cell types such as dopaminergic neurons. The CAGE method we used detected gene regulatory changes at the level of promoters, providing additional information compared to transcript level-detection in regular RNA sequencing methods. We observed caffeine dose-dependent increases in the up-regulation of IEG expression, in terms of the number of promoters, number of genes, as well as fold change levels. IEG expression changes were detected even from cell culture manipulation as shown by the comparison of control cells, in which no caffeine was added. Most previous studies have used relatively high dosages (mM concentrations) to study the effects of caffeine, likely due to the concern that lower concentrations of caffeine may not induce measurable changes. Our study tested micromolar caffeine concentrations, representing consumption levels. We showed that even 3 μM of caffeine increased the number of IEGs detected compared to the control. This observation emphasizes the importance of experimental design when only small effects might be expected. Often studies only have negative controls at 0 h; in our study, we also sampled the no caffeine control samples at all time points during the time course, which allowed us to detect gene expression fluctuations that happen normally in cells with minimal perturbations.

We investigated the expression levels of adenosine receptors. Consumption levels of caffeine mainly have antagonistic effects on A_1,_ A_2A_ and A_2B_ in the brain. A_2B_ requires higher concentrations of adenosine for activation and therefore is presumed to be less important for caffeine antagonism^1^. Since A_2A_ was not appreciably expressed in our neuronal cell model, the gene expression changes we observed were likely due to blocking of A_1_ actions. Previous microarray study in the mouse striatum^30^ identified A_2A_-dependent and A_2A_-independent pathways affected by caffeine, as well as different gene sets differentially expressed from low vs. high doses of caffeine. To identify genes and pathways regulated by A_2A_, a different NES cell differentiation protocol for developing dopaminergic neurons would be needed, as A_2A_ is mainly expressed in dopamine-rich regions of the brain^1^.

The effects of caffeine on axon growth and neuronal projections have previously been studied at the macro level^24^. Our study identified multiple genes perturbed in these pathways that could act as biomarkers in future molecular studies. The novel genes and pathways in response to caffeine treatment identified in our study could have been activated due to and/or in addition to caffeine’s inhibition of adenosine binding to adenosine receptors. Caffeine appeared to have its inherent regulatory effect on gene expression towards increasing neuronal cell connectivity by regulating genes for axon growth and more generally neuron projection development. Although the results were due to acute effects of caffeine treatment, it would be interesting to see if these pathways are also up-regulated with prolonged exposure to caffeine. If the activation of neuronal connection/development pathways could be observed in a long term treatment model, it might help explain the links between caffeine and improved cognitive performance, as well as suggested protective effects against Alzheimer disease in epidemiological studies^41–43^.

Since the relationships of some of the over-represented GO biological processes in the set of genes down-regulated by 10 μM caffeine treatment were not obvious to us at first glance, a literature search was conducted to identify other studies possibly supporting our findings. We found that in other studied systems such as chicken and rat neurons, caffeine indeed inhibited axon growth^44, 45^. In human myeloid leukemic cells and stem cell differentiation to adipocytes, extracellular matrix expression was modified or down-regulated^46, 47^ upon caffeine treatment. Inhibition of wound healing was seen in primary keratinocytes after 4 days of caffeine treatment^48^. As for the regulation of immune system process, the anti-inflammatory effect of caffeine has been studied using immune cells in mice, rats, as well as human whole blood^49^. Although the time frame, caffeine concentration and study systems were very different from our study, it appears that the down-regulation effects of these processes have been observed previously in multiple studies. However, our study is the first to show down-regulation of immune system process regulatory genes at such doses of caffeine.

Our study only looked at low doses of caffeine. Due to limited number of replicates providing insufficient power combined with the rather small difference between the concentrations tested, we did not observe direct dosage-dependent effects for the up-regulated genes, where higher concentration induced higher gene expression fold changes. By using more replicates and testing more caffeine dosages, we would expect to identify more genes with direct dosage-dependent expression profiles. One complicating factor is that caffeine often produces biphasic responses, such that very low doses and very high doses have neutral or depressing effects, whereas medium dosages have more stimulatory effects^1, 50^. A likely explanation is that caffeine can influence multiple molecular targets. The potency of caffeine at these target(s) appears to be dependent on many factors, including age of individual, hormone status, genetics (e.g. CYP1A2 polymorphism), previous drug intake, and more^51^. Repeated consumption of caffeine can lead to tolerance to some but not all effects^1^. The level of adenosine as well as the number of adenosine receptors may also change with repeated caffeine consumption. Since the depressive physiological effects induced by high dosages of caffeine are not due to antagonism of adenosine receptors^50^, one could use such (perhaps supraphysiological) dosages of caffeine to identify pathways activated that are distinct from the ones activated by adenosine receptor antagonism.

The fact that caffeine can be metabolized by the liver within 1 h adds another layer of complexity in the understanding molecular mechanisms of caffeine’s effects on the brain^52^. The main metabolites of caffeine include paraxanthine, theophylline, and theobromine^53^, which are more potent adenosine receptor antagonists than caffeine^54^. They produce overlapping but some different effects on the body^50^. In a cell culture model, only caffeine is present to act on the neurons. In the human body, the total magnitude of the adenosine receptor blockade would include the trimethylxantine caffeine as well as the dimethylxanthine metabolites. Each metabolite could be tested individually to elucidate their independent effects on neuronal pathways.

In conclusion, we have used a nonmalignant human neuronal-glial cell culture model and an RNA profiling method that tags transcript start sites to study the effect of caffeine on neurons, yielding increased resolution compared to previous methods. Our results replicated and extended previous observations on caffeine-induced up-regulation of immediate early genes. We observed short-term down-regulation by caffeine on neuronal projection inhibition, as well as stimulatory effects of neuronal projection, suggesting promotion of neuronal connections that might provide mechanistic insights into the enhancing effects of caffeine on memory and cognition.

## Materials and Methods

### NES cells

The culturing of human long-term self-renewing neuroepithelial stem cells (NES cells, line AF22) and ethical guidelines were described in Falk *et al*.^31^. In brief, cells were cultured on plates pre-coated with poly-L-ornithine (0, 1 mg/ml; Sigma) and laminin (2 µg/ml; Sigma). NES cells were grown in DMEM/F12 + GLUTAMAX supplemented with Penicillin (100 U/ml) and Streptomycin (100 µg/ml), N2 (1:100; Life Technologies), B27 (1:1000; Life Technologies), EGF (10 ng/ml; Peprotech), FGF (10 ng/ml; life technologies) and the medium was changed every day. To induce differentiation, cells were initially plated in complete medium. After 2 days, the medium was changed to medium without growth factors EGF and FGF. After 7 days, the medium was changed to a 1:1 ratio mixture of Neurobasal medium (Life Technologies) supplemented with Glutamax (Life Technologies) and DMEM/F12 + GLUTAMAX, supplemented with N2 (1:200) and B27 (1:100). NES cells were differentiated for 38 days after removal of growth factors and half of the medium was changed every 2–3 days containing laminin (1:1000). At time point 0, the medium was changed to medium containing caffeine at the concentrations 3 µM and 10 µM (and 0 µM) and cells were collected 0 h, 1 h, 3 h and 9 h later using NucleoSpin RNA kit (Macherey-Nagel). At least three replicates were collected for each condition.

### Immunofluorescence

Cells were fixed in 4% formaldehyde (Sigma). The fixed cells were blocked and permeabilized in 5% horse serum (Life Technologies), 0.05% PBS-Tween for 1 h at room temperature and incubated o/n at 4 °C with primary antibody (anti-Map2, Abcam, ab5392, 1:500; anti-NeuN, Millipore, ABN78, 1:200; anti-GFAP, R&D systems, AF2594, 1:2000; anti-βIII-tubulin (anti-TUJ1), Covance, MMS-435P, 1:400). Cells were incubated for 1 h at room temperature with secondary antibodies (Alexa Goat anti-chicken 488, Invitrogen, A11039, 1:1000; Alexa donkey anti-rabbit 568, Invitrogen, A10042, 1:1000; Alexa donkey anti-mouse 568, Invitrogen, A10037, 1:1000; Alexa donkey anti-sheep 488, Invitrogen, A11015; 1:1000). Nuclei were stained with DRAQ5 (Cell Signaling Technology) at 1:1000 for 10 min at room temperature. Images were acquired on an A1R Ti confocal (Nikon Instruments, Inc.) using a Plan Apo λ 10 × NA 0.45 objective in z-stack acquisition mode, 1024 × 1024 pixel size and 3 × 12 bit color space. Images were processed using Nikon NIS-elements version 4.51 (Laboratory Imaging/Nikon). Maximum intensity projection was applied and DRAQ5 was pseudo-colored in blue. Images were modified by applying LUT and converted to 8 bit RGB, then exported as tiff files.

### RNA extraction and CAGE Library preparation

Total RNA was extracted from NES cell cultures using NucleoSpin RNA kit (Macherey-Nagel) according to manufacturer’s instructions. RNA concentrations were measured using Nanodrop ND-1000 (Thermo fisher scientific) and the RNA quality was checked using Bioanalyzer RNA pico kit (Agilent technologies). CAGE libraries were prepared with total RNA input of 1 μg as described previously^55^. Linker dimer contaminations were processed with E-gel 2% starter kit (Invitrogen) according to manufacturer’s instructions. Individual samples were barcoded such that four CAGE libraries could be pooled for one sequencing lane. To minimize batch effects, libraries were randomized such that replicates and samples processed in the same week were in different pools. Sequencing was performed on an Illumina HiSeq. 2500 (Illumina).

### RNA sequence processing and quality check

The FastX toolkit (http://hannonlab.cshl.edu/fastx_toolkit/) was used for preprocessing steps^56^. Only reads with >50% of the bases with quality score 30 were used. Reads were trimmed to 25 bases from the 12^th^ base using Fastq trimmer. Bowtie 1.0.1^57^ was used to map the reads to human genome version hg19. SAMTools^58^ was used to convert alignment files between SAM and BED formats. Overlapping reads, or CAGE initial transcription start sites (CTSS), were clustered into tag clusters (TC) by Paraclu v.9^59^. Read counts were normalized to tags per million (TPM). CTSS with less than 0.1 TPM were excluded from the clustering step. TCs with <30 counts or length >200 bps were also excluded. 43,900 TCs mapped to hg19 were obtained. Gencode v19^60, 61^ was used to annotate the TC, resulting in 14,018 annotated genes. Expression values for these TCs were computed by using BedTools^62^ to intersect the unfiltered CTSS files with the Paraclu-determined TC genomic boundaries.

### Gene expression analysis

Gene expression differential analysis was performed for samples from each time point and each caffeine concentration compared to 0 h controls with no caffeine added. In addition, for each individual time point, differential analysis was performed for the three caffeine concentrations. DESeq 2^63^ was used for all the gene expression differential analyses, with adjusted p-value < 0.05 and a fold change cut-off set at ±1.5 fold (log_2_ FC = 0.58). Reads mapped to Ensembl gene annotations (release 75) were considered as transcript start sites (TSS). Gene ontology (GO) enrichment analysis was performed using ToppGene^64^, with P-value cut off set at 0.05 adjusted by the Benjamini-Hochberg procedure. The list of 232 reference immediate-early response genes was obtained from Supplementary Table S5 from Arner *et al*. study (https://figshare.com/articles/Supplementary_figures_tables_and_texts_for_FANTOM_5_phase_2/1288777)^65^. The averaged time course progression was calculated using the mean expression fold changes compared to control at time 0 for the set of genes within a selected over-represented GO biological process.

### Data availability

The aligned CAGE RNA-Seq BAM files generated during the current study are available at the European Nucleotide Archive (ENA) repository, project number PRJEB20092.

## Electronic supplementary material

Supplementary Information

Table S1

Table S2

Table S3
