# Supplementary material for: Acute doses of caffeine shift nervous system cell expression profiles toward promotion of neuronal projection growth
Source: Sci Rep. 2017 Sep 13;7:11458. doi: 10.1038/s41598-017-11574-6 (PMC5597620; doi:10.1038/s41598-017-11574-6)

Acute doses of caffeine shift nervous system cell expression profiles toward promotion of neuronal projection growth

Nancy Y. Yu[1], Andrea Bieder[1], Amitha Raman[2], Enrichetta Mileti[1], Shintaro Katayama[1], Elisabet Einarsdottir[1,3], Bertil B. Fredholm[4], Anna Falk[5], Isabel Tapia-Páez[1,†], Carsten O. Daub[1,6], Juha Kere[1,3,7*]

1. Department of Biosciences and Nutrition, Karolinska Institutet, Huddinge, Sweden.

2. Department of Medicine (MedH), Karolinska Institutet, Huddinge, Sweden.

3. Folkhälsan Institute of Genetics, and Molecular Neurology Research Program, University of Helsinki, Helsinki, Finland.

4. Department of Physiology and Pharmacology, Karolinska Institutet, Solna, Sweden.

5. Department of Neuroscience, Karolinska Institutet, Solna, Sweden.

6. Division of Genomic Technologies, RIKEN Center for Life Science Technologies, Tsurumi, Yokohama, Japan.

7. Department of Medical and Molecular Genetics, King's College London, London, United Kingdom.

* Corresponding author, e-mail: juha.kere@ki.se

†Present affiliation: Department of Medicine/Center for Molecular Medicine, Karolinska University Hospital, Solna, SE-171 76, Sweden.

Supplementary Information

Supplementary Table S1. Lists of differentially expressed TSS and their annotations for all time points and caffeine concentrations compared to 0 μM caffeine control at time 0, shown in a separate Excel file.

Supplementary Table S2. Lists of annotated TSS differentially expressed at 3 h between treatments of different caffeine concentrations - 10 μM vs. 0 μM, 10 μM vs. 3 μM, and the list of TSS common to the two comparisons, shown in a separate Excel file.

Supplementary Table S3. ToppGene GO enrichment lists for a) differentially expressed genes for 10 μM caffeine treatment at 3 h compared to control at 0 h, b) caffeine induced dosage-dependent down-regulated genes at 3 h, and c) caffeine induced dosage-dependent up-regulated genes, shown in a separate Excel file.

Supplementary Figure S1. NES cells were differentiated for 38 days (no treatment with caffeine) and immunolabeled with glial cell marker GFAP and neuronal cell marker βIII-TUB (TUJ1). Nuclei were labeled with DRAQ5. The majority of cells are labeled with βIII-TUB, while a small number of cells are positive for GFAP.

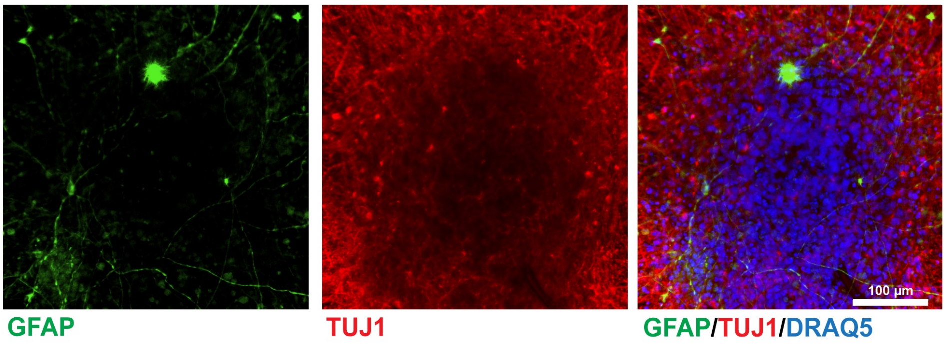

Supplementary Figure S2. CAGE RNA Expression levels for ADORA1 (A1) TSS1, ADORA1 TSS2, and ADORA2B displayed for all time course samples treated with 0 μM (red), 3 μM (green), and 10 μM (blue) of caffeine. The expression levels are shown as mean TPM±1 s.d. for 0 h, 1 h, 3 h and 9 h.

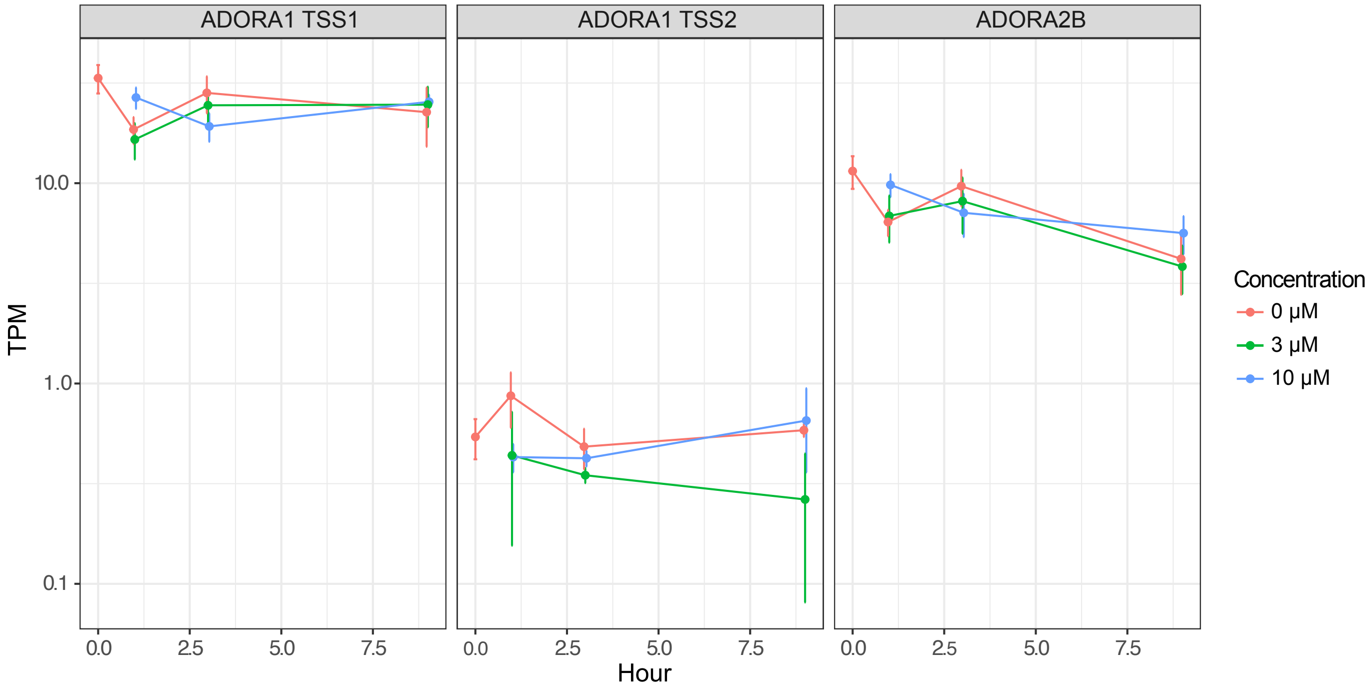

Supplement: Supplementary file 1 — Supplementary Information [file 41598_2017_11574_MOESM1_ESM.pdf]
